# Bouncing back, if not beyond: Challenges for research on resilience

**DOI:** 10.1057/s41291-020-00133-z

**Published:** 2020-09-11

**Authors:** Martin Hoegl, Silja Hartmann

**Affiliations:** 1 Institute for Leadership and Organization, Ludwig-Maximilians-Universität München, Geschwister-Scholl-Platz 1, 80539 Munich, Germany; 2 Chair of Organization, Freie Universität Berlin, Garystr. 21, 14195 Berlin, Germany

**Keywords:** Resilience, Setbacks, Positive adaptation, Cross-level, Events, Cross-cultural, Inter-cultural

## Abstract

Setbacks are a fact of life for individuals and collectives—and resilience is a key concept in explaining why some entities positively adapt (i.e., bounce back) or even emerge stronger (i.e., bounce beyond), while others suffer from such events, sometimes permanently. In this short note, we briefly introduce the concept of resilience before moving to three key challenges for management research in this field. With this, we would like to encourage the international scholarly research community to view any phenomenon of their interest also from a resilience perspective, considering significant setbacks and processes of positive adaptation.

## Introduction

No individual, no team, no organization, no nation, or collective of any type simply always rushes from one success to the next. Instead, setbacks and the experience of adverse circumstances are a fact of live as humans and larger systems consistently face a multitude of internal and external life prompts, stressors, opportunities, and other forms of change (Richardson 2002). With regard to organizational contexts, this is amplified against the backdrop of increasingly dynamic business environments, such as in China or India, as well as cross-national interdependencies and business relationships within Asia and with the rest of the world. Management research addresses this reality from at least two main perspectives, risk management and resilience (Fiksel et al. 2015; van der Vegt et al. 2015). The notion of risk management caters, more or less implicitly, to the idea of risks being something that can be assessed ex ante and aims at creating protective measures commensurate to the risk assessment. The concept of resilience differs from this logic in several important ways.

First, the term resilience refers to “positive adaptation within the context of significant adversity” (Luthar et al. 2000: p. 543) and is therefore less concerned with questions of risk predictions and prevention, but rather starts its analysis with a significant setback event. Such setbacks can take various shapes and forms, ranging from individuals’ setbacks, such as being passed over once again for a career promotion or teams’ setbacks, such as a major work project being prematurely terminated due to strategy changes by upper management, all the way to large-scale company or industry setbacks, such as the ‘Diesel-Gate’ scandal of 2015, that first plunged Volkswagen into disarray before spreading to large parts of the automobile industry. More precisely, the definition of resilience encompasses ‘significant adversity’, implying that such setbacks endanger the functioning of a system (individuals, teams, organizations, etc.) severely and fundamentally (Hartmann et al. 2020a; Masten 2001; van der Vegt et al. 2015). And it is that severity that helps us distinguish between setbacks that call for resilience versus stressors that triggers regular coping responses (Richardson 2002). Such severity, however, lies in the eye of the beholder and the availability of protective factors at their disposal (Bonanno et al. 2015). What constitutes significant adversity for us may well be a more minor disturbance to other people.

Second, resilience, as the definition by Luthar et al. (2000) points out, is concerned with positive adaptation following such a significant setback. As such, the question is not about how to avoid setbacks—as risk management would—, but what to do after one hit. Here lies an important aspect of resilience that distinguishes it from phenomena such as regular stress coping. While regular stress typically prompts various coping responses that individuals, teams or larger collectives have established over time, significant setbacks are qualitatively different from such stress in that individuals, teams, and larger collectives do not have preset responses for positive adaptation. Whether this is due to negligent risk management, a general carelessness, or a deliberate decision not to take any preparatory measures, given that low event probabilities make such precautions economically questionable, the setback in question has occurred and is experienced as significant by the affected entity. The question of how to proceed from here is what is key in the resilience process.

Given this general framing of resilience, the purpose of this paper is to briefly discuss three key challenges of resilience research, including a special focus on a better cross-cultural understanding of this phenomenon. Before we introduce and discuss these three challenges, we consider it helpful to provide a backdrop on the state of the art in terms of our understanding of resilience as a concept.

## The resilience process

**Fig. 1:**
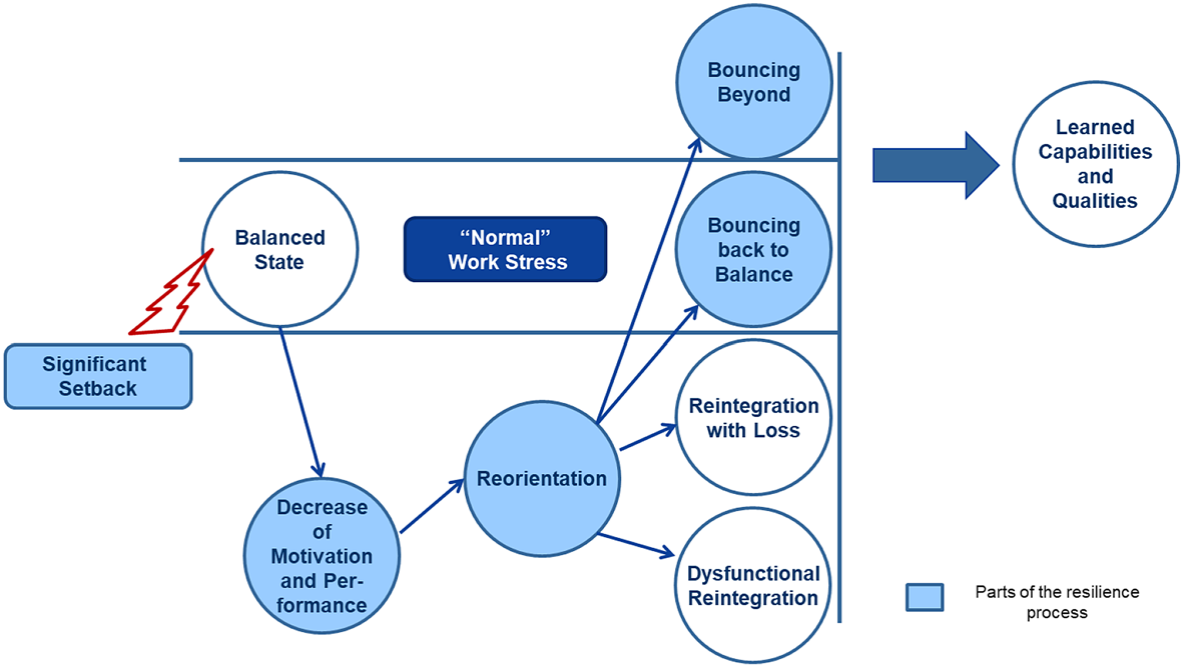
The resilience process, adapted from Richardson (2002), p. 311

Resilience research has a tradition in many different disciplines, such as psychology (Masten 2001), socioecology (Holling 1973), or engineering (Hollnagel et al. 2007). We draw on the psychological perspective and conceptualize resilience as a process, which is the most current and perhaps the most encompassing view on the phenomenon (Williams et al. 2017). Drawing on Richardson (2002) and adapted to a management context, Fig. 1 illustrates this understanding and shows how a significant setback can literally knock an entity out of bounds, leading to a decrease of motivation or/and performance, followed by a period of reorientation, where ways toward a positive adaptation are potentially being identified and put into action. This phase of reorientation, then, can yield various outcomes. A non-resilient response would yield reintegration with losses, e.g., a permanent decrease in motivation, where, for instance, product development engineers become permanently less committed and more risk averse in their innovation endeavors as a result of another premature project termination. A resilient response, by contrast, would be one where, after a certain time of reorientation, an entity bounces back to its pre-setback state. And of course, a significant setback can also provide an opportunity for development and growth, whereby individuals, teams, or larger collectives emerge stronger and more capable than before—what some have termed ‘bouncing beyond’—and eventually learn new skills and capabilities.

This process conceptualization illustrates two important aspects of resilience as a phenomenon. First, it integrates different perspectives on resilience, e.g., capacity and outcome approaches, that have been discussed in the literature. As such, resilience can be seen as an outcome (Britt et al. 2016; DesJardine et al. 2019), or as a general potential (Moenkemeyer et al. 2012; Stoverink et al. 2018). In our example, bouncing back or beyond is the manifestation of a resilient response. However, resilience can also be attributed as a property to entities such as individuals, teams, or larger collectives, particularly if they have successfully completed a resilience process or exhibit so-called resilience potential (i.e., possess characteristics that make it likely for them to fare well if and when significant adversity hits). In addition, the process perspective allows to shed light on cognitive, affective, and behavioral mechanisms that happen in the face of adversity and which may enable or restrain entities to achieve a resilient outcome. In our example, these responses are a form of a reorientation. Thus, second, resilience seen as a process helps to focus managerial attention on both the adversity and the positive adaptation that needs to follow (Hartmann et al. 2020a). In other words, a process perspective allows to investigate both defining elements of resilience and their interplay.

Against this very brief background, we now turn our attention to what we see as three major challenges for resilience research, attention to which is necessary to improve our understanding of how entities deal with unforeseen setbacks, the likes of which are all around us.

## Three challenges

### Challenge 1: From individual to collective resilience—cross-level influences

One strong tradition in resilience research originates from psychology and has thus focused on individuals’ resilience. This dominant focus on the individual is still very present in current scholarly activity in management (King et al. 2016), investigating employees’ individual resilience (e.g., Green et al. 2011; Shin et al. 2012). Yet, management scholars have also shed light on resilience at the organizational level (Kahn et al. 2018; Ortiz-de-Mandojana and Bansal 2016). In this regard, a strong stream of research has been formed around high reliability organizations (Sutcliffe 2011; Weick and Sutcliffe 2001; Weick et al. 1999). In contrast, comparatively little research attention has been devoted to the resilience of teams (Hartmann et al. 2020b; Stoverink et al. 2018), which can be regarded as a link between individual-level behaviors and organizational-level processes.

To create a coherent understanding of resilience at different levels of analysis and, specifically, of the resilience of larger collectives, such as industries, communities, and economies, we will need to consider both the collective as well as the sub-collective levels as an integrated system within which interdependent resilience processes unfold at various levels. For example, concerning this interdependence, studying the ‘Black Saturday’ bushfires, a 2009 series of bushfires that burned across the Australian state of Victoria, Shepherd and Williams (2014) argued that local venturing helped connecting local individuals with the abundant resources of a broader community. Despite this research and a few other important studies (e.g., Carmeli and Markman 2011; Rao and Greve 2018; Williams and Shepherd 2016), we know very little as to what makes communities (such as the city Port-au-Prince after the devastating Haiti earthquake disaster of 2010), entire industries (such as the aviation industry in the wake of the Coronavirus crisis), or regional economies (such as the European Union in the face of large-scale migration) adapt positively in the face of significant adversity. Importantly, interdependent resilience processes may lead to different outcomes for different (sub-)entities. For instance, with the onset of a global crises such as the one caused by the coronavirus (SARS-CoV-2), the response of social distancing from other people is likely to lead to resilience on a societal level by flattening the infection curve, yet it might reduce citizens’ individual resilience to positively cope with the crisis as these humans may suffer from social isolation. We know very little about these cross-level influences of multiple resilience processes within the context of an adverse event (Hartmann et al. 2020b). Further, in many cases larger collectives are not affected by adversity as a whole, but rather in parts. Concerning organizations, at first only single departments, teams, or hierarchical levels may face mounting demands, which overwhelm capacities and in consequence transform to an adverse situation for the whole organization (Kahn et al. 2018). For instance, a hospital emergency department may become overcrowded, which may not affect other parts of the hospital at first. However, over time the hospital emergency department may be forced to focus on only the most critical patients while increasingly drawing on staff and resources from other departments, which may reduce patient care and result in diminished overall hospital effectiveness. A nested view on resilience within social systems that acknowledges relations among different parts and analytical levels is key, to unlock the more complex patterns (e.g., crossover effects, and upward or downward spirals) of how some systems adapt more positively than others. Theoretical lenses, such as conservation of resources theory, social identity theory, or group relations theory (Hobfoll et al. 2018; Hogg et al. 2012; Tajfel and Turner 1979; Voronov and Vince 2012), may help to explicate and explain these complex patterns.

### Challenge 2: Taking events seriously, not just features and structures

Research on resilience has focused quite strongly on characteristics of individuals (and to a smaller degree, on collectives) that make them more or less vulnerable in the face of significant adversity. Resilience research in the management and organizational sciences has quite willingly carried this approach forward, as explaining variance across entities based on features, structures, and other (stable) characteristics of individuals, teams, and organizations has a strong tradition in our field. It is certainly worthwhile explaining the resilience of teams through team characteristics such as relationship quality and communication, and to do so via statistical analyses across a large sample. While such inquiry continues to be important, we advocate—based more on a process view of resilience—to also take events seriously. In this regard, event systems theory (Morgeson et al. 2015) provides a theoretical lens and defines events as discrete occurrences bounded in time and space. Perhaps the most important events in analyses of resilience processes are the significant setbacks. We advocate putting them front and center in our inquiries. Events system theory suggests various aspects of ‘event strength’, such as novelty (indicated through the absence of established scripts or routines to guide action), disruption (indicated by the event blocking or transforming ongoing routines), and criticality (indicated through the attention and resource allocation the event commands) (Morgeson et al. 2015). Even though event system theory defines events as bounded in time and space, in many cases initial events may form larger chains of events that multilaterally affect each other. Similarly, it is possible that a specific event creates anxieties that remain and impede functioning over a longer period of time (Barton and Kahn 2019). Thus, even though an event is bounded in time, its potentially adverse consequences may persist longer. As such, the actions of individuals, companies, or industries as events unfold are likely to be reciprocally interdependent. One fitting, albeit extreme, example of this is the coronavirus crisis with its intertwined humanitarian and economic effects spreading throughout Asia and beyond. However, a focus on events also implies that we need to consider how these events are formed in the first place. Less disruptive, but unexpected occurrences may cause changes that lead to chronic stressors or accumulate into pending crisis events, which may then trigger the need for resilience (Fisher et al. 2019; Williams et al. 2017). Overall, we know very little about how the characteristics of specific adverse events as well as their formation and unfolding affect downstream activities and subsequent events. Yet, it is likely that relations among variables of interest will differ depending on the type or the experience of adversity (Gucciardi et al. 2018). For instance, how larger collectives frame an adverse event (internally or externally) may determine cooperative action and thus collective resilience (Rao and Greve 2018). We consider it important to better understand how the nature of the setback events and its specific consequences determines downstream practices of positive adaptation.

### Challenge 3: Resilience across and between cultures

As with most management research, also resilience scholarship shows a bias toward research with cultural majorities in western nations—with a lack of studies in Asian contexts. For instance, measures of resilience primarily reflect a westernized selection of items (Ungar 2013; Windle et al. 2011). While this is a shortcoming of the field in general, it is particularly lamentable in the case of resilience, given that extant research suggest that culture and contextual embeddedness may play a role in determining resilience (Pangallo et al. 2015; Ungar 2013). For example, studies have found that cultural orientations that emphasize sociability may foster individual resilience, whereas cultural values that promote self-reliance seem to be related to symptoms of burn-out (Wei and Taormina 2014; Welbourne et al. 2015). Further, members of cultures that are better attuned to ecological conditions might be better able to sense changes in ecological conditions and adapt to these (Whiteman and Cooper 2011). Beyond these influences, even though there are universal mechanisms that seem to promote resilience across cultures, such as access to supportive relationships, it is likely that some resilience processes might not play out the same way across cultures (Ungar 2013). Thus, researchers have suggested that a culturally and contextually embedded understanding of resilience is needed (Ungar 2008), which also holds implications for management research.

For instance, the meaning of what establishes an outcome of positive adaptation may vary to some degree across cultures that differ regarding values, beliefs, and everyday practices (Masten and Coatsworth 1998). Also, we know very little about the framing and attribution of setbacks across different regions and cultures, even though setback and failure accounts are likely to vary (e.g., Cardon et al. 2011). Similarly, learning from setbacks (i.e., bouncing beyond), a central theme (if not to say mantra) across the start-up scene of North America, may not necessarily transfer to Asian or other cultural contexts and be just as effective there. While start-ups certainly face significant setbacks across the world, how individuals and collectives in such places as Paris, Johannesburg, Singapore, and Beijing deal with them likely differs. And what if teams are internationally mixed? Given the likely differences of resilience processes across cultural contexts, and the increasing cultural diversity in modern workplaces and organizations (Molinsky 2007; Pieterse et al. 2013), inter-cultural settings become all the more relevant domains of inquiry. These questions are pertinent certainly beyond the context of start-ups that tend to (at least at first) often be rather ‘domestic’ in nature. Yet how does resilience unfold in truly multi-national or global companies, where size, diversity, and age come much more strongly into play? Acknowledging the cultural and contextual embeddedness of the concept of resilience will also help to highlight that even though individuals, teams, or organizations may promote their levels of resilience, whether an entity will succeed to positively adapt after experiencing a significant setback is largely dependent on the respective systemic structures and opportunities for resource access, which is why the responsibility for resilience may not solely be placed on the individual (or team or organization).

## Conclusion

Resilience remains a concept of utmost importance. We suspect that every reader will agree that the long-term success of any entity (individual or collective) is likely to depend not only on successes, but also on dealing with setbacks. Yet management research around the world seems much more inclined to study what is commonly seen as ‘success factors’ such as strategy, leadership, and innovation, perhaps unintentionally neglecting the less obvious long-term driver of performance and well-being that we discussed here. In closing, therefore, we would like to invite the international scholarly research community to view any phenomenon of their interest also from a resilience perspective, considering significant setbacks and processes of positive adaptation.
